# Systemic inflammation and insulin resistance-related indicator predicts poor outcome in patients with cancer cachexia

**DOI:** 10.1186/s40170-024-00332-8

**Published:** 2024-01-25

**Authors:** Guo-Tian Ruan, Li Deng, Hai-Lun Xie, Jin-Yu Shi, Xiao-Yue Liu, Xin Zheng, Yue Chen, Shi-Qi Lin, He-Yang Zhang, Chen-An Liu, Yi-Zhong Ge, Meng-Meng Song, Chun-Lei Hu, Xiao-Wei Zhang, Ming Yang, Wen Hu, Ming-Hua Cong, Li-Chen Zhu, Kun-Hua Wang, Han-Ping Shi

**Affiliations:** 1 Department of Gastrointestinal Surgery/Department of Clinical Nutrition, Beijing Shijitan Hospital, Capital Medical University, 10 Tie Yi Road, Beijing, 100038 China; 2 National Clinical Research Center for Geriatric Diseases, Xuanwu Hospital, Capital Medical University, 10 Tie Yi Road, Beijing, 100053 China; 3 Key Laboratory of Cancer FSMP for State Market Regulation, 10 Tie Yi Road, Beijing, 100038 China; 4 Laboratory for Clinical Medicine, Capital Medical University, 10 Tie Yi Road, Beijing, 100038 China; 5 Clinical Nutrition Department, Sichuan University West China Hospital, Chengdu, 610041 Sichuan China; 6 Comprehensive Oncology Department, National Cancer Center/Cancer Hospital, Chinese Academy of Medical Sciences and Peking Union Medical College, Beijing, 100038 China; 7 Department of Immunology, School of Preclinical Medicine, Guangxi Medical University, Nanning, 530021 China; 8 Yunnan University, Kunming, 650091 China; 9 General Surgery Clinical Medical Center of Yunnan Province, Kunming, 650032 China

**Keywords:** Systemic inflammation, Insulin resistance, CTI, Overall survival

## Abstract

**Background:**

The C-reactive protein (CRP)-triglyceride-glucose (TyG) index (CTI), which is a measure representing the level of inflammation and insulin resistance (IR), is related to poor cancer prognosis; however, the CTI has not been validated in patients with cancer cachexia. Thus, this study aimed to explore the potential clinical value of the CTI in patients with cancer cachexia.

**Methods:**

In this study, our prospective multicenter cohort included 1411 patients with cancer cachexia (mean age 59.45 ± 11.38, 63.3% male), which was a combined analysis of multiple cancer types. We randomly selected 30% of the patients for the internal test cohort (mean age 58.90 ± 11.22% 61.4% male). Additionally, we included 307 patients with cancer cachexia in the external validation cohort (mean age 61.16 ± 11, 58.5% male). Receiver operating characteristic (ROC) and calibration curves were performed to investigate the prognostic value of CTI. The prognostic value of the CTI was also investigated performing univariate and multivariate survival analyses.

**Results:**

The survival curve indicated that the CTI showed a significant prognostic value in the total, internal, and external validation cohorts. Prognostic ROC curves and calibration curves revealed that the CTI showed good consistency in predicting the survival of patients with cancer cachexia. Multivariate survival analysis showed that an elevated CTI increased the risk of death by 22% (total cohort, 95% confidence interval [CI] = 1.13–1.33), 34% (internal test cohort, 95%CI = 1.11–1.62), and 35% (external validation cohort, 95%CI = 1.14–1.59) for each increase in the standard deviation of CTI. High CTI reliably predicted shorter survival (total cohort, hazard ratio [HR] = 1.45, 95%CI = 1.22–1.71; internal test cohort, HR = 1.62, 95%CI = 1.12–2.36; external validation cohort, HR = 1.61, 95%CI = 1.15–2.26). High CTI significantly predicted shorter survival in different tumor subgroups, such as esophageal [HR = 2.11, 95%CI = 1.05–4.21] and colorectal cancer [HR = 2.29, 95%CI = 1.42–3.71]. The mediating effects analysis found that the mediating proportions of PGSGA, ECOG PS, and EORTC QLQ-C30 on the direct effects of CTI were 21.72%, 19.63%, and 11.61%, respectively We found that there was a significant positive correlation between the CTI and 90-day [HR = 2.48, 95%CI = 1.52–4.14] and 180-day mortality [HR = 1.77,95%CI = 1.24–2.55] in patients with cancer cachexia.

**Conclusion:**

The CTI can predict the short- and long-term survival of patients with cancer cachexia and provide a useful prognostic tool for clinical practice.

**Supplementary Information:**

The online version contains supplementary material available at 10.1186/s40170-024-00332-8.

## Background

According to the cancer burden statistics of GLOBOCAN for 2020, there are an estimated 19.3 million new cancer cases and nearly 10 million cancer deaths worldwide [1]. Cancer cachexia is a multifactorial syndrome defined as decreased appetite, weight, and skeletal muscle [2], resulting in fatigue [3], functional impairment [4], increased treatment-related toxicity [5], poor quality of life [6], and reduced survival [7]. Abnormalities associated with cancer cachexia include changes in carbohydrate, lipid, and protein metabolism as well as increased anorexia, insulin resistance (IR), and muscle protein degradation [8]. This is driven by a combination of reduced food intake (due to apparent anorexia) and increased energy consumption caused by high metabolic states [9]. Notably, the degree of cancer cachexia depends on the tumor type and tumor stage. For example, the prevalence of cachexia is about 70% in pancreatic cancer and 30% or less in other types of cancer, such as breast and prostate cancer [10]. Additionally, cancer treatments, including chemotherapy and radiotherapy, can also lead to cachexia syndrome [11]. In cancer, 50% of patients develop this syndrome; as the condition worsens, the quality of life, treatment tolerance, treatment response, and survival rate decrease, and the prevalence rate increases to 80% [12].

Systemic inflammation and IR play important roles in cancer cachexia. Systemic inflammation in cachexia arises from numerous sources, including tumor cells, tumor-infiltrating cells, parenchymal cells of the surrounding tissue, and related infiltrating cells [13–15]. Pro-inflammatory cytokines secreted by these cells include tumor necrosis factor (TNF)-α, interleukin (IL)-6, and IL-1β, and many studies have focused on the characteristics of cachexia induced by these factors [14, 15]. IR occurs in patients with cancer and even in patients with cancer cachexia [16]. In patients with cancer cachexia, increased endogenous glucose production, gluconeogenesis (GNG), and IR have been observed; however, unlike in type 2 diabetes (T2D), fasting blood glucose (FBG) levels are within the normal range [17]. In colon-26 tumor mice, IR was found in the early stages of cachexia before weight loss [18]. In patients with sarcoma without significant weight loss, intravenous glucose tolerance tests showed impaired glucose tolerance in patients with lower body weight [19]. Severe malnutrition or weight loss in cancer patients accompanies decreased insulin levels [20]. Chronic inflammation in patients with large weight loss can lead to pancreatic cell dysfunction and impaired insulin secretion [21].

Systemic inflammation and IR are intertwined, and the interaction between them may predict poor prognosis. Elevated level of C-reactive protein (CRP) indicates a system inflammation response [22]. In previous reports, CRP was found to be independently associated with insulin insensitivity as a predictor of cardiovascular events [23]. Both the primary tumor itself and the related inflammatory response cause cytokine production, and CRP production also increases [24]. Thus, CRP may be used as an indicator of tumor recurrence [25, 26]. A multi-cancer study found that IR was associated with systemic inflammation in patients [27]. Patients with cancer are exposed to pro-inflammatory cytokines and insulin growth factor binding proteins, which leads to cancer cachexia [28] and results in IR [29]. Cytokines may damage the insulin signaling pathway by phosphorylating the insulin receptor and its substrate [30]. Xia et al. found that inflammation is important in the occurrence of IR via the immune system [31]. IR was associated with CRP levels in moderate weight loss in 10 male patients with non-small cell lung cancer [17]. In patients with cancer, CRP levels in the circulatory system are elevated [32]. Wigmore et al. found that the level of inflammation decreased after resection of tumor tissue in 202 patients with colorectal cancer, indicating that the existence of a primary tumor is directly or indirectly related to the production of CRP [24]. Previous studies have reported peripheral IR in patients with non-small cell lung [17], gastrointestinal [33], and colorectal cancer [34].

Currently, the fasting triglyceride and glucose levels (labeled as the TyG index) is considered as a simple measure tool of IR in many tumor-related studies [35–37]. In our previous study, we developed a new indicator of inflammatory insulin resistance indicator, the CRP-TyG index (CTI), which can better predict the prognosis of patients with cancer. Because inflammation and insulin resistance are closely related to cancer cachexia, and inflammation and insulin resistance are related to the survival and treatment of cancer cachexia, this study is based on the previously established inflammation and insulin related index -CTI, which can reflect the level of inflammation and insulin resistance, and predict the survival of patients with cancer cachexia.

## Methods

### Data source and selection criteria

This was a prospective cross-sectional observational study using data from the INSCOC (Investigation on Nutrition Status and its Clinical Outcome of Common Cancers) cancer and patient nutrition project [38–45], which collected data from hospitals or clinics in multiple regions of China from 2013 to 2021. In the present study, 4697 patients with cancer from the INSCOC cohort were included. In addition, to validate the constructed prognostic index, we also collected the data from a cohort of patients with cancer at the Zhejiang Cancer Hospital for external validation. Patient inclusion criteria were as follows: (1) pathological diagnosis of cancer, (2) 18 years of age, and (3) normal consciousness and no communication barrier. No strict exclusion criteria were applied. This study was approved by the research ethics committees of the respective medical centers. All patients provided written informed consent prior to the interview.

In our study, the data collected were based on hospital medical records and face-to-face questionnaires. The baseline characteristics collected in this study include sex, age, body mass index (BMI), tumor stage, tumor types, undergo surgery (yes/no), undergo radiotherapy (yes/no), undergo chemotherapy(yes/no), smoking status (yes/no), alcohol consumption (yes/no), diabetes (yes/no), hypertension (yes/no), coronary heart disease (yes/no), Karnofsky Performance Status (KPS), The European Organization for Research and Treatment of Cancer-Quality of Life Questionnaire-Core 30 (EORTC QLQ-C30), Eastern Cooperative Oncology Group Performance Status (ECOG PS), Patient-Generated Subjective Global Assessment (PGSGA), receive nutritional intervention (yes/no), and triceps skinfold thickness (TSF). Tumor stages were defined and classified according to the 8th edition of the TNM system. The BMI was calculated as weight (kg) / height (m^2^). The classification of BMI was based on the standards of the Chinese population: < 18.5; 18.5–24.9; 25–28, and > 28 kg/m^2^. Blood samples were collected by professional nurses within 8 h of fasting or within 48 h of fasting before treatment. The laboratory indicators included C-reactive protein (CRP), fasting blood glucose (FBG), total cholesterol (TC), and triglyceride levels. The triglyceride-glucose (TyG) index was calculated using the following formula: ln [TG (mg/dl) × FBG (mg/dl]) /2. In this study, the inflammation-IR index constructed and developed was the CRP-TyG index (CTI), calculated as follows: CTI = 0.412 × ln (CRP) + TyG [41].

### Diagnosis of cancer cachexia

The definition and assessment of cancer cachexia followed the diagnostic criteria of Fearon in 2011 [9]: (1) Unintentional weight loss of > 5% in the past 6 months; (2) BMI < 20 kg / m^2^ and weight loss > 2%; (3) Loss of skeletal muscle mass (sarcopenia) and weight loss of > 2%. Skeletal muscle loss was assessed by anthropometry (male 32 cm^2^, female 18 cm^2^) to determine the middle and upper arm muscle areas [9]. After cachexia diagnosis evaluation, 1411 patients with cancer were assessed for cancer cachexia in the multicenter cohort, while 307 patients with cancer were assessed for cancer cachexia in the external validation cohort [see Additional file 1].

### Follow-up and endpoint assessment

The follow-up records for this study were obtained by telephone consultation and from annual hospital follow-ups from the time of the first hospitalization to the diagnosis of cancer. The primary observation endpoint of this study was overall survival (OS). OS was defined as the time from the initial diagnosis of cancer to the death of the participant or the date of the last follow-up. In addition, the secondary end events observed in this study were 90-day and 180-day mortality, which were defined as deaths from the beginning of the study to the 90-day and 180-day follow-ups.

### Statistical analysis

In this study, continuous variables satisfying normal distribution were reported by mean plus or minus standard deviation, and the t-test was used for comparisons between groups. Continuous variables that did not meet the normal distribution were expressed as median plus or minus quartile, and the Wilcoxon test was used for comparisons between groups. Categorical variables are reported as numbers and percentages, and the chi-square test was used to compare categorical variables. We performed Pearson correlation analysis, and it is considered that there is a significant correlation between variables when the correlation coefficient is greater than 4 or less than-4 and the statistical *P*-value is less than 0.05. In this study, the optimal cut-off value of CTI in patients with cancer cachexia was determined by the maximum selection rank statistics, and the optimal cut-off value of CTI in patients with cancer cachexia was 4.71 [see Additional file 2]. The patients were classified into four categories according to the quartile of CTI (Q), and the CTI of Q1 was < 4.20, Q2 was 4.20 ~ 4.62, Q3 was 4.62 ~ 5.20, and Q4 was > 5.20. The patients were classified into three categories according to the quartile of CTI (T), and the CTI of T1 was < 4.33, T2 was 4.33 ~ 5.00, and T3 was > 5.00. In the multicenter cohort, we randomly selected 30% of 1411 patients with cancer cachexia as the internal verification cohort. The details are presented in the flowchart [see Additional file 2]. Finally, the multivariate Cox regression survival analysis of all three cohorts were performed to determine the prognostic value of CTI in patients with cachexia. To further reduce the interference of confounding factors and determine the prognostic value of the CTI, we constructed different adjustment models: model 0, unadjusted; model 1, adjusted for sex, age, and BMI; and model 2, adjusted for sex, age, BMI, tumor stage, tumor type, surgery, chemotherapy, radiotherapy, smoking status, alcohol consumption, KPS, EORTC QLQ-C30, ECOG PS, PGSGA, nutritional intervention, diabetes, hypertension, and coronary heart disease. Model 3 was adjusted for sex, age, BMI, tumor stage, tumor type, KPS, surgery, chemotherapy, radiotherapy, smoking status, alcohol consumption, KPS, EORTC QLQ-C30, ECOG PS, PGSGA, nutritional intervention, diabetes, hypertension, coronary heart disease, and TSF. Hazard ratios (HRs) and 95% confidence intervals (CI) were performed to evaluate univariate and multivariate Cox survival analysis. Furthermore, the prognostic receiver operating characteristic (ROC) and calibration curves were constructed and developed to evaluate the short- and long-term survival prediction ability and consistency of the CTI in the multicenter total, internal test, and external verification cohorts to determine the prognostic value of the CTI in patients with cancer cachexia. In addition, univariate and multivariate logistic regression analyses were also performed to evaluate the association between CTI and the risk of 90-day and 180-day mortality. Odds ratios (ORs) and 95% CI were used for the logistic regression analysis.

All analyses were performed using R, version 4.0.3. A *P*-value < 0.05 (two-tailed) was considered to be statistically significant, except *P* < 0.1 in the interaction test.

## Results

### Baseline characteristics

In this study, 1411 patients with cancer cachexia were included in the multicenter cohort, including 420 patients with cancer cachexia in internal test cohort. Additionally, 307 patients with cancer cachexia were included in external validation cohort [see Additional file 1]. The baseline characteristics of the three cohorts are shown in Table 1. In multicenter cohort, the average age of patients with cancer cachexia was 59.45 ± 11.38 years, including 894 (63.3%) males, and the average CTI was 4.68 ± 0.72. In internal test cohort, the average age of patients with cancer cachexia was 58.90 ± 11.22 years, including 258 (61.4%) males, and the average CTI was 4.72 ± 0.72. In external validation cohort, the average age of patients with cancer cachexia was 61.16 ± 11.58 years, including 192 (62.5%) males, and the average CTI was 4.69 ± 0.77.

**Table 1:** Baseline characteristics of this study population

Variables	Total cohort	Internal test cohort	External validation cohort	*p*
	(*n* = 1412)	(*n* = 420)	(*n* = 307)	
Sex (%)				0.776
Male	894(63.3)	258(61.4)	192(62.5)	
Female	518(36.7)	162(38.6)	115(37.5)	
Age (mean (SD))	59.45(11.38)	58.90(11.22)	61.16(11.58)	0.023
BMI (mean (SD))	21.02(3.19)	21.27(3.31)	20.15(2.84)	< 0.001
Tumor stage (%)				< 0.001
I	83(5.9)	25(6.0)	6(2.0)	
II	232(16.4)	59(14.0)	26(8.5)	
III	416(29.5)	128(30.5)	83(27.0)	
IV	681(48.2)	208(49.5)	192(62.5)	
Tumor types (%)				0.005
Lung cancer	382(27.1)	111(26.4)	73(23.8)	
Gastric cancer	314(22.2)	79(18.8)	72(23.5)	
Other digestive cancers	119(8.4)	33(7.9)	31(10.1)	
Esophageal cancer	129(9.1)	44(10.5)	23(7.5)	
Colorectal cancer	277(19.6)	79(18.8)	67(21.8)	
Breast cancer	46(3.3)	19(4.5)	5(1.6)	
Female reproductive cancer	49(3.5)	21(5.0)	11(3.6)	
Urological cancer	29(2.1)	14(3.3)	3(1.0)	
Nasopharyngeal cancer	34(2.4)	9(2.1)	2(0.7)	
Other cancer	33(2.3)	11(2.6)	20(6.5)	
Surgery, yes (%)	684(48.4)	217(51.7)	167(54.4)	0.122
Radiotherapy, yes (%)	136(9.6)	44(10.5)	36(11.7)	0.522
Chemotherapy, yes (%)	801(56.7)	242(57.6)	195(63.5)	0.091
Tch, mmol/L (mean (SD))	4.44(1.16)	4.43(1.15)	4.37(1.17)	0.651
TG (mean (SD))	1.33(0.83)	1.37(1.01)	1.28(0.73)	0.355
TyG (mean (SD))	3.82(0.28)	3.83(0.29)	3.86(0.29)	0.137
CRP (mean (SD))	25.10(41.99)	26.11(42.00)	25.01(37.09)	0.901
CTI (mean (SD))	4.68(0.72)	4.72(0.72)	4.69(0.77)	0.62
Glucose (mean (SD))	5.66(1.75)	5.74(2.04)	6.31(2.15)	< 0.001
Smoking, yes (%)	711(50.4)	205(48.8)	123(40.1)	0.005
Drinking, yes (%)	353(25.0)	112(26.7)	88(28.7)	0.378
Diabetes, yes (%)	139(9.8)	48(11.4)	27(8.8)	0.476
Hypertension, yes (%)	255(18.1)	79(18.8)	83(27.0)	0.001
CHD, yes (%)	66(4.7)	16(3.8)	6(2.0)	0.088
KPS (mean (SD))	82.83(14.77)	83.52(13.87)	74.40(15.16)	< 0.001
QC30 (mean (SD))	50.38(13.50)	50.09(13.61)	57.26(16.41)	< 0.001
ECOG PS (%)				< 0.001
< 2	1234 (87.4)	370 (88.1)	200 (65.1)	
≥ 2	178 (12.6)	50 (11.9)	107 (34.9)	
PGSGA (%)				0.682
Well-nourished	55 (3.9)	19 (4.5)	10 (3.3)	
Malnutrition	1357 (96.1)	401 (95.5)	297 (96.7)	
Nutrition intervention, yes (%)	332 (23.5)	99 (23.6)	185 (60.3)	< 0.001
TSF (mean (SD))	13.53 (6.82)	13.57 (7.11)	20.54 (15.73)	< 0.001

*CTI* CRP-TyG index, *CRP* C-reactive protein, *TyG* triglyceride-glucose index, *BMI* body mass index, *CHD* coronary heart disease, *KPS* karnofsky performance status, *EORTC QLQ-C30* The European Organization for Research and Treatment of Cancer (EORTC), Quality of Life Questionnaire-Core 30 (QLQ-C30), *ECOG PS* eastern cooperative oncology group performance status, *PGSGA* Patient Generated Subjective Global Assessment, *TSF* triceps skinfold thickness

### Survival analysis of CTI in the total cohort, internal test cohort, and external validation cohort

**Fig. 1:**
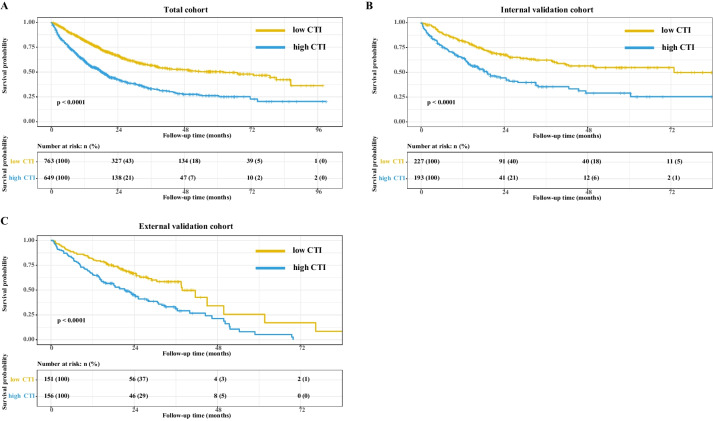
The Kaplan–Meier survival curves of CTI in the different cohorts of patients with cancer cachexia. **A** Total cohort; **B** Internal test cohort; **C** External validation cohort. The "yellow line" represents patients with cancer cachexia with low CTI, and the "blue line" represents patients with cancer cachexia with high CTI. Notes: CTI, C-reactive protein-triglyceride glucose index

**Fig. 2:**
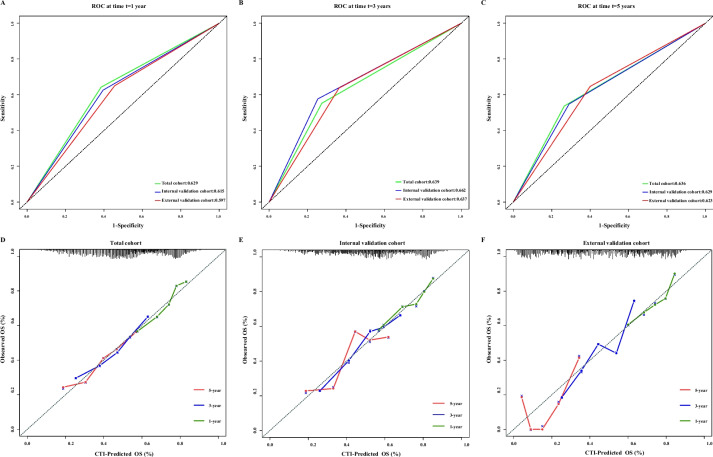
The 1-, 3-, and 5-year prognostic ROC and calibration curves of CTI in the different cohorts of patients with cancer cachexia. **A**-**C** 1-, 3-, and 5-year prognostic ROC curves, **A** Total cohort, **B** Internal test cohort, **C** External validation cohort; **D**-**F** 1-, 3-, and 5-year prognostic calibration curves, **D** Total cohort, **E** Internal test cohort, **F** External validation cohort. Notes: CTI, C-reactive protein-triglyceride glucose index; ROC, receiver operating characteristic

Figure 1 shows the survival curves relative to the CTI in the total cohort, internal test cohort, and external validation cohort, suggesting that patients with cancer cachexia with a high CTI had a poorer survival than those with a low CTI (all *P* < 0.001). The results of cumulative survival analysis are consistent with those in Fig. 1 [see Additional file 3]. Figure 2 shows that the HR of patients increased with an increase in the CTI, which showed consistent results in the total, internal validation, and external validation cohorts.

In total cohort, we performed a multivariate survival analysis showed that when CTI was used as a continuous variable, each SD increase in the CTI reflected increased death risk in patients with cancer cachexia by 22% (after adjusting model 3, 95%CI = 1.13–1.33, *P* < 0.001). When CTI was used as a binary variable, high CTI in patients with cancer cachexia predicted worse survival (HR = 1.45, 95%CI = 1.22–1.71, *P* < 0.001). When CTI scores were classified into four categories, the risk of death increased significantly compared with group Q3 (HR = 1.48, 95%CI = 1.17–1.88, *P* = 0.001) and group Q4 (HR = 1.76, 95%CI = 1.38–2.24, *P* < 0.001) and showed an increasing trend with the risk of death (*P* for trend < 0.001). When CTI was classified into three categories, the risk of death increased significantly compared with group T2 (HR = 1.40, 95%CI = 1.14–1.73, *P* = 0.001) and group T3 (HR = 1.60, 95%CI = 1.30–1.97, *P* < 0.001), and showed an increasing trend with the risk of death (*P* for trend = 0.002). It is worth noting that we observed consistent results in both internal and external validation cohorts and that CTI is a good survival indicator for patients with cancer cachexia (Table 2).

**Table 2:** Survival analyses

Variables	OS (model 0) ^a^		OS (model 1) ^b^		OS (model 2) ^c^		OS (model 3) ^d^	
	Crude HR(95%CI)	Crude P	Adjusted HR(95%CI)	Adjusted P	Adjusted HR(95%CI)	Adjusted P	Adjusted HR(95%CI)	Adjusted P
**Total cohort**								
As continues (per SD)	1.48 (1.37–1.59)	< 0.001	1.49 (1.39–1.61)	< 0.001	1.21 (1.12–1.31)	< 0.001	1.22 (1.13–1.33)	< 0.001
By cut-off								
CTI < 4.71	ref		ref		ref		ref	
CTI ≥ 4.71	2.12 (1.81–2.47)	< 0.001	2.12 (1.82–2.48)	< 0.001	1.44 (1.22–1.70)	< 0.001	1.45 (1.22–1.71)	< 0.001
By Interquartile								
Q1	ref		ref		ref		ref	
Q2	1.29 (1.01–1.64)	0.041	1.35 (1.06–1.73)	0.015	1.23 (0.96–1.58)	0.101	1.25 (0.97–1.60)	0.081
Q3	1.99 (1.59–2.50)	< 0.001	2.02 (1.61–2.54)	< 0.001	1.48 (1.16–1.87)	0.001	1.48 (1.17–1.88)	0.001
Q4	2.82 (2.26–3.52)	< 0.001	2.93 (2.34–3.67)	< 0.001	1.73 (1.36–2.20)	< 0.001	1.76 (1.38–2.24)	< 0.001
P for trend		< 0.001		< 0.001		< 0.001		< 0.001
By tertiles								
T1	ref		ref		ref		ref	
T2	1.56 (1.28–1.91)	< 0.001	1.60 (1.31–1.96)	< 0.001	1.41 (1.15–1.74)	0.001	1.40 (1.14–1.73)	0.001
T3	2.42 (2.00–2.93)	< 0.001	2.46 (2.02–2.98)	< 0.001	1.60 (1.30–1.96)	< 0.001	1.60 (1.30–1.97)	< 0.001
P for trend		< 0.001		< 0.001		< 0.001		< 0.001
**Internal test cohort**								
As continues (per SD)	1.46 (1.25–1.70)	< 0.001	1.45 (1.24–1.69)	< 0.001	1.35 (1.12–1.63)	0.002	1.34 (1.11–1.62)	0.002
By cut-off								
CTI < 4.71	ref		ref		ref		ref	
CTI ≥ 4.71	1.96 (1.42–2.69)	< 0.001	1.90 (1.37–2.65)	< 0.001	1.64 (1.12–2.39)	0.01	1.62 (1.12–2.36)	0.011
By Interquartile								
Q1	ref		ref		ref		ref	
Q2	1.62 (0.96–2.76)	0.072	1.36 (0.79–2.36)	0.268	1.22 (0.68–2.17)	0.504	1.22 (0.69–2.18)	0.495
Q3	2.32 (1.42–3.78)	0.001	2.08 (1.26–3.42)	0.004	1.83 (1.08–3.12)	0.026	1.83 (1.08–3.11)	0.026
Q4	2.83 (1.77–4.52)	< 0.001	2.58 (1.60–4.17)	< 0.001	2.03 (1.17–3.53)	0.012	2.01 (1.16–3.48)	0.013
P for trend		< 0.001		< 0.001		0.005		0.006
By tertiles								
T1	ref		ref		ref		ref	
T2	1.83 (1.19–2.81)	0.005	1.63 (1.05–2.51)	0.029	1.37 (0.86–2.20)	0.186	1.39 (0.87–2.24)	0.169
T3	2.61 (1.74–3.91)	< 0.001	2.43 (1.61–3.69)	< 0.001	1.97 (1.22–3.16)	0.005	1.93 (1.21–3.10)	0.006
P for trend		< 0.001		< 0.001		0.005		0.006
**External validation cohort**								
As continues (per SD)	1.50 (1.30–1.73)	< 0.001	1.48 (1.28–1.71)	< 0.001	1.32 (1.12–1.55)	0.001	1.35 (1.14–1.59)	< 0.001
By cut-off								
CTI < 4.71	ref		ref		ref		ref	
CTI ≥ 4.71	2.17 (1.61–2.92)	< 0.001	2.12 (1.57–2.87)	< 0.001	1.59 (1.13–2.24)	0.007	1.61 (1.15–2.26)	0.006
By Interquartile								
Q1	ref		ref		ref		ref	
Q2	1.53 (0.94–2.46)	0.084	1.67 (1.03–2.70)	0.039	2.31 (1.37–3.89)	0.002	2.30 (1.36–3.88)	0.002
Q3	1.97 (1.23–3.16)	0.005	2.00 (1.25–3.22)	0.004	2.15 (1.26–3.66)	0.005	2.16 (1.27–3.70)	0.005
Q4	3.59 (2.30–5.61)	< 0.001	3.65 (2.33–5.73)	< 0.001	3.24 (1.94–5.40)	< 0.001	3.28 (1.97–5.47)	< 0.001
P for trend		< 0.001		< 0.001		< 0.001		< 0.001
By tertiles								
T1	ref		ref		ref		ref	
T2	1.46 (0.98–2.18)	0.064	1.50 (1.00–2.24)	0.05	1.72 (1.11–2.68)	0.016	1.71 (1.10–2.67)	0.017
T3	2.74 (1.89–3.99)	< 0.001	2.67 (1.83–3.89)	< 0.001	2.42 (1.56–3.76)	< 0.001	2.44 (1.57–3.79)	< 0.001
P for trend		< 0.001		< 0.001		< 0.001		< 0.001

*OS* overall survival, *HR* hazards ratio, *CI* confidence interval, *CTI* CRP-TyG index, *CRP* C-reactive protein, *TyG* triglyceride-glucose index, *BMI* body mass index, *KPS* karnofsky performance status *EORTC QLQ-C30* The European Organization for Research and Treatment of Cancer (EORTC), Quality of Life Questionnaire-Core 30 (QLQ-C30), *ECOG PS* eastern cooperative oncology group performance status, *PGSGA* Patient Generated Subjective Global Assessment, *TSF* triceps skinfold thickness

^a^Model 0: Unadjusted

^b^Model 1: Adjusted for age, sex, and BMI

^c^Model 2: Adjusted for age, sex, BMI, tumor stage, tumor types, surgery, chemotherapy, radiotherapy, smoking status, alcohol consumption, KPS, EORTC QLQ-C30, ECOG PS, PGSGA, diabetes, hypertension, and coronary heart disease

^d^Model 3: Adjusted for age, sex, BMI, tumor stage, tumor types, KPS, surgery, chemotherapy, radiotherapy, smoking status, alcohol consumption, KPS, EORTC QLQ-C30, ECOG PS, PGSGA, diabetes, hypertension, coronary heart disease, and TSF

**Fig. 3:**
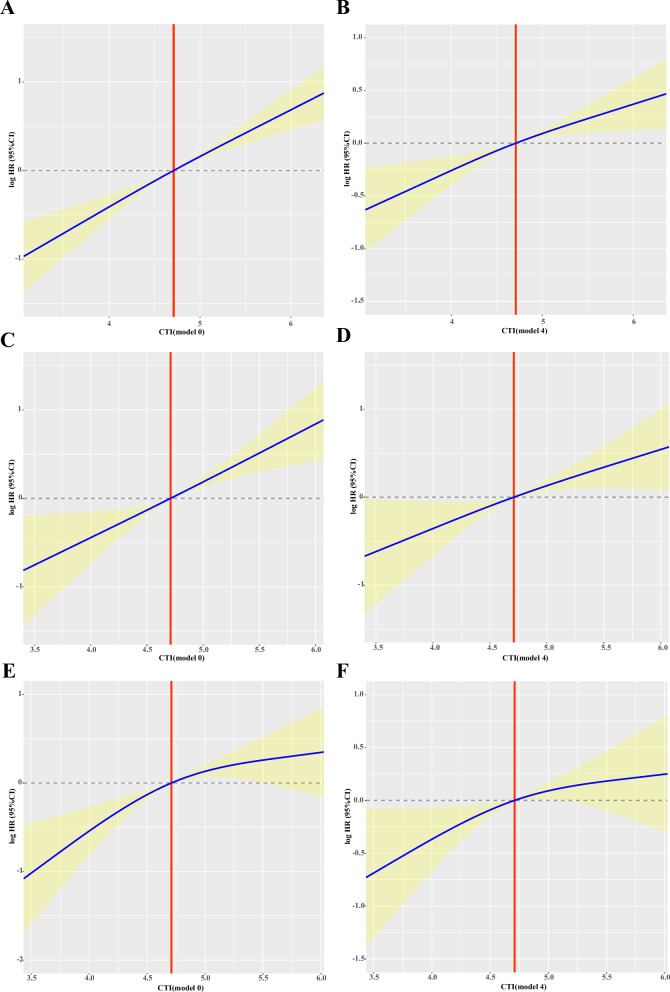
The restricted cubic spline curves of CTI in the different cohorts of patients with cancer cachexia. **A**, **B** Total cohort, **A** Unadjusted, **B** Adjusted for model 4; **C**, **D** Internal test cohort, **C** Unadjusted, **D** Adjusted for model 4; **E**, **F** External validation cohort, **E** Unadjusted, **F** Adjusted for model 4; Model 4: adjusted for age, sex, BMI, tumor stage, tumor type, KPS, surgery, chemotherapy, radiotherapy, smoking status, alcohol consumption, KPS, EORTC QLQ-C30, ECOG PS, PGSGA, diabetes, hypertension, coronary heart disease, and TSF. Notes: CTI, C-reactive protein-triglyceride glucose index; BMI: body mass index; KPS, karnofsky performance status; EORTC QLQ-C30, The European Organization for Research and Treatment of Cancer (EORTC), Quality of Life Questionnaire-Core 30 (QLQ-C30); ECOG PS: eastern cooperative oncology group performance status; PGSGA, Patient Generated Subjective Global Assessment; TSF, triceps skinfold thickness

We generated prognostic ROC curves showed that CTI had better survival prediction ability at 1 year (total cohort: 0.629; internal test cohort: 0.615; external validation cohort: 0.597), 3 years (total cohort: 0.639; internal test cohort: 0.662; external validation cohort: 0.637) and 5 years (total cohort: 0.636; internal test cohort: 0.629; external validation cohort: 0.623) (Fig. 3A-C). In addition, the 1-year, 3-year, and 5-year calibration curve results showed that the CTI had a good ability to predict short-term and long-term survival in patients with cancer cachexia, whether in the total cohort, internal test cohort, or external validation cohort (Fig. 3D-F).

### Baseline characteristics stratified by CTI

As previously described, we investigated and determined the prognostic value of the CTI in patients with cancer cachexia in the total cohort, internal test cohort, and external validation cohort. Therefore, our follow-up analysis was based on the total cohort data. Patients with cancer cachexia were classified into high CTI and low CTI groups. The baseline characteristics stratified by CTI showed that patients with high CTI were more likely to be men and older adults, with higher tumor stages, lower KPS scores, higher EORTC QLQ-C30 scores, more ECOG PS ≥ 2 scores, and malnourished patients (Table 3).

**Table 3:** Baseline characteristics stratified by CTI

Variables	CTI < 4.71	CTI ≥ 4.71	*P*-value
	(*n* = 763)	(*n* = 649)	
Sex (%)			0.066
Male	466(61.1)	428(65.9)	
Female	297(38.9)	221(34.1)	
Age (mean (SD))	58.27(11.66)	60.84(10.88)	< 0.001
BMI (mean (SD))	20.89(3.07)	21.17(3.31)	0.105
Tumor stage (%)			< 0.001
I	52(6.8)	31(4.8)	
II	174(22.8)	58(8.9)	
III	255(33.4)	161(24.8)	
IV	282(37.0)	399(61.5)	
Tumor.type (%)			< 0.001
Lung cancer	169(22.1)	213(32.8)	
Gastric cancer	212(27.8)	102(15.7)	
Other digestive cancers	53(6.9)	66(10.2)	
Esophageal cancer	76(10.0)	53(8.2)	
Colorectal cancer	157(20.6)	120(18.5)	
Breast cancer	28(3.7)	18(2.8)	
Female reproductive cancer	19(2.5)	30(4.6)	
Urological cancer	12(1.6)	17(2.6)	
Nasopharyngeal cancer	26(3.4)	8(1.2)	
Other cancer	11(1.4)	22(3.4)	
Surgery, yes (%)	418(54.8)	266(41.0)	< 0.001
Radiotherapy, yes (%)	62(8.1)	74(11.4)	0.047
Chemotherapy, yes (%)	418(54.8)	383(59.0)	0.122
Tch, mmol/L (mean (SD))	4.46(1.04)	4.41(1.29)	0.396
TG (mean (SD))	1.15(0.49)	1.53(1.07)	< 0.001
CRP (mean (SD))	3.64(3.05)	50.33(51.47)	< 0.001
CTI (mean (SD))	4.14(0.41)	5.32(0.42)	< 0.001
Glu (mean (SD))	5.33(1.14)	6.05(2.21)	< 0.001
Smoking, yes (%)	358(46.9)	353(54.4)	0.006
drinking, yes (%)	196(25.7)	157(24.2)	0.558
diabetes, yes (%)	45(5.9)	94(14.5)	< 0.001
hypertension, yes (%)	115(15.1)	140(21.6)	0.002
CHD, yes (%)	29(3.8)	37(5.7)	0.119
KPS (mean (SD))	85.54(11.42)	79.63(17.39)	< 0.001
QC30 (mean (SD))	47.85(12.87)	53.36(13.62)	< 0.001
ECOG PS (%)			< 0.001
< 2	712(93.3)	522(80.4)	
≥ 2	51(6.7)	127(19.6)	
PGSGA (%)			0.003
Well-nourished	8.89(3.92)	10.81(4.67)	
Malnutrition	722(94.6)	635(97.8)	
Nutrition intervention, yes (%)	160(21.0)	172(26.5)	0.017
TSF (mean (SD))	13.52(6.64)	13.53(7.03)	0.978

*CTI* CRP-TyG index, *CRP* C-reactive protein, *TyG* triglyceride-glucose index, *BMI* body mass index, *CHD* coronary heart disease, *KPS* karnofsky performance status, *EORTC QLQ-C30* The European Organization for Research and Treatment of Cancer (EORTC), Quality of Life Questionnaire-Core 30 (QLQ-C30), *ECOG PS* eastern cooperative oncology group performance status, *PGSGA* Patient Generated Subjective Global Assessment, *TSF* triceps skinfold thickness

### Distribution of CTI in different subgroups

**Fig. 4:**
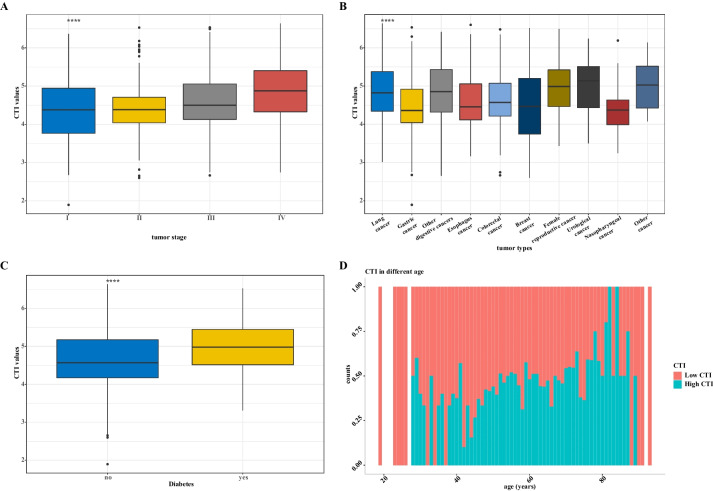
The distribution of CTI in different groups. **A** CTI in tumor stage groups; **B** CTI in tumor type groups; **C** CTI in diabetes and non-diabetes groups; **D** CTI in different age groups

As shown in Fig. 4, the distribution curves found that the higher the CTI value, the greater the tumor progression. The distribution of different tumor types showed that there were relatively low CTI levels in patients with gastric, breast, and nasopharyngeal cancers and relatively high CTI levels in patients with lung, female reproductive system, and urological cancers. As expected, the CTI was higher in patients with diabetes than in those without diabetes. Notably, the proportion of patients with high CTIs increased with age [see Additional file 4].

### Sensitivity analysis and subgroup analysis

After removing the information of patients who died within 3 months, the sensitivity analysis showed that CTI showed a good ability to predict survival, whether as a continuous or categorical variable, which was consistent with the previous description [see Additional file 5]. We performed survival analysis in different tumor subgroups, and after multivariate adjustment, we observed that high CTI predicted worse survival in esophageal cancer (HR = 2.11; 95CI = 1.05–4.21; *P* = 0.035) and colorectal cancer (HR = 2.29; 95CI = 1.42–3.71; *P* = 0.001) (Table 4).

**Table 4:** Survival analysis in different tumor types

Variables	OS (model 0)		OS (model 3)	
	Crude HR(95%CI)	Crude P	Adjusted HR(95%CI)	Adjusted P
Lung cancer				
CTI < 4.71	ref		ref	
CTI ≥ 4.71	1.51 (1.16–1.97)	0.002	1.22 (0.93–1.61)	0.151
Esophagus cancer				
CTI < 4.71	ref		ref	
CTI ≥ 4.71	2.64 (1.61–4.34)	< 0.001	2.11 (1.05–4.21)	0.035
Gastric cancer				
CTI < 4.71	ref		ref	
CTI ≥ 4.71	1.70 (1.21–2.4)	0.002	1.28 (0.86–1.9)	0.221
Colorectal cancer				
CTI < 4.71	ref		ref	
CTI ≥ 4.71	3.24 (2.15–4.89)	< 0.001	2.29 (1.42–3.71)	0.001
Female tumor				
CTI < 4.71	ref		ref	
CTI ≥ 4.71	3.02 (1.32–6.92)	0.009	1.51 (0.49–4.59)	0.472
Other cancer				
CTI < 4.71	ref		ref	
CTI ≥ 4.71	2.47 (1.63–3.73)	< 0.001	1.82 (1.15–2.89)	0.011

Model 0: Unadjusted

Model 3: Adjusted for age, sex, BMI, tumor stage, KPS, surgery, chemotherapy, radiotherapy, smoking status, alcohol consumption, KPS, EORTC QLQ-C30, ECOG PS, PGSGA, diabetes, hypertension, coronary heart disease, and TSF

*OS* overall survival, *HR* hazards ratio, *CI* confidence interval, *CTI* CRP-TyG index, *CRP* C-reactive protein, *TyG* triglyceride-glucose index, *BMI* body mass index, *KPS* karnofsky performance status, *EORTC QLQ-C30* The European Organization for Research and Treatment of Cancer (EORTC), Quality of Life Questionnaire-Core 30 (QLQ-C30), *ECOG PS* eastern cooperative oncology group performance status, *PGSGA* Patient Generated Subjective Global Assessment, *TSF* triceps skinfold thickness

**Fig. 5:**
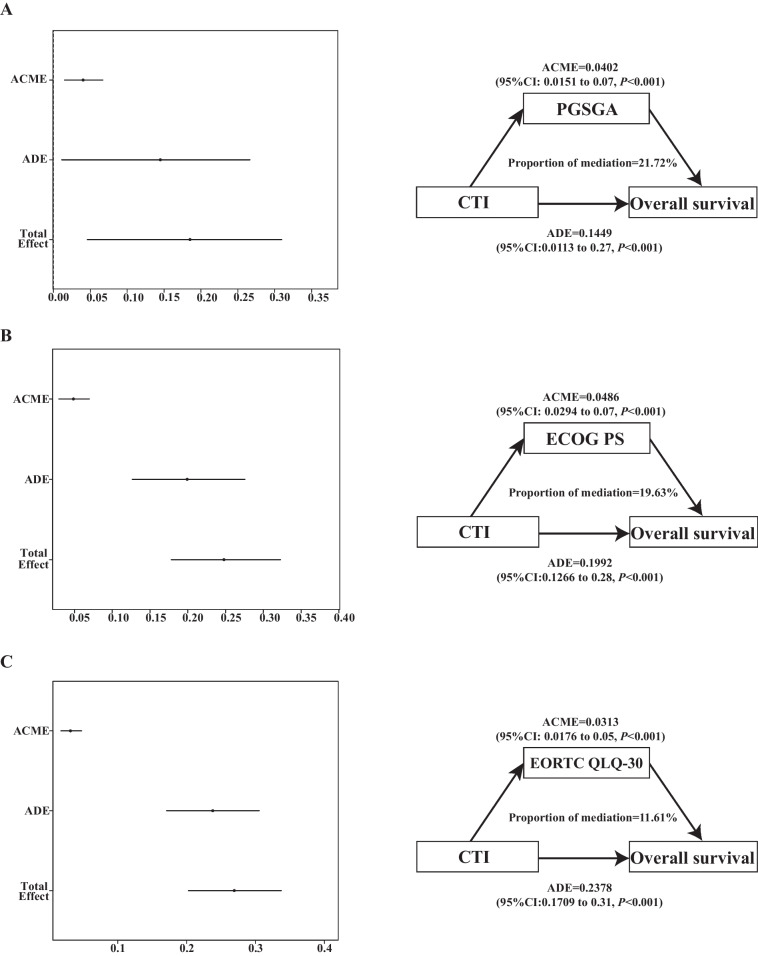
The mediation proportion of PGSGA, ECOG PS, and EORTC QLQ-C30 in CTI attributed to OS in patients with cancer cachexia. **A** PGSGA; **B** ECOG PS; **C** EORTC QLQ-C30. Notes: CTI, C-reactive protein-triglyceride glucose index; PGSGA, Patient Generated Subjective Global Assessment; ECOG PS, eastern cooperative oncology group performance status; EORTC QLQ-C30, The European Organization for Research and Treatment of Cancer (EORTC), Quality of Life Questionnaire-Core 30 (QLQ-C30); OS, overall survival

Our subgroup analysis found a significant interaction between the CTI and patients undergoing surgery (*P* = 0.068) and radiotherapy (*P* = 0.069) (Fig. 5).

### Mediation analyses

**Fig. 6:**
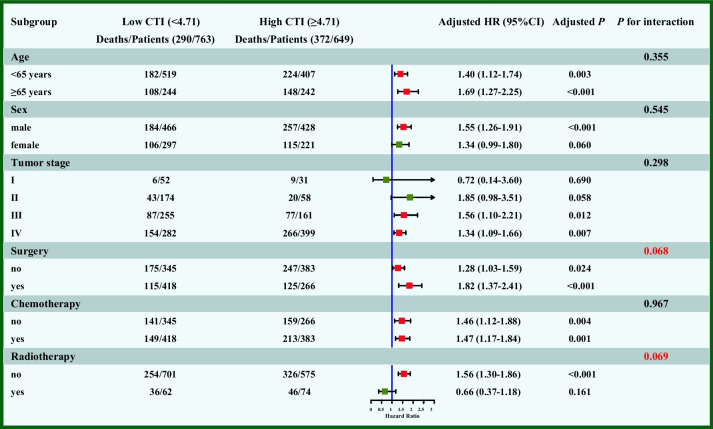
The subgroup analysis of the CTI in the total cohort of patients with cancer cachexia. Adjusted for age, sex, BMI, tumor stage, tumor type, KPS, surgery, chemotherapy, radiotherapy, smoking status, alcohol consumption, KPS, EORTC QLQ-C30, ECOG PS, PGSGA, diabetes, hypertension, coronary heart disease, and TSF. Notes: CTI, C-reactive protein-triglyceride glucose index; BMI: body mass index; KPS, karnofsky performance status; EORTC QLQ-C30, The European Organization for Research and Treatment of Cancer (EORTC), Quality of Life Questionnaire-Core 30 (QLQ-C30); ECOG PS: eastern cooperative oncology group performance status; PGSGA, Patient Generated Subjective Global Assessment; TSF, triceps skinfold thickness

As shown in Fig. 6, we investigated the mediating effects and found that the mediating proportions of PGSGA, ECOG PS, and EORTC QLQ-C30 on the direct effects of CTI were 21.72%, 19.63%, and 11.61%, respectively.

### Association of CTI with 90-day and 180-day mortality risk

Additional file 6 shows the association between CTI and 90-day and 180-day mortality risk in patients with cancer cachexia. We observed that there was a significant positive correlation between CTI and the risk of 90-day (OR = 2.48, 95%CI = 1.52–4.14, *P* < 0.001) and 180-day (OR = 1.77, 95%CI = 1.24–2.55, *P* < 0.001) mortality in patients with cancer cachexia.

## Discussion

In this study, the CTI was an effective survival predictor reflecting the inflammatory and IR states of patients with cancer cachexia, and based on the results of prognostic ROC and calibration curves, the CTI could predict the short-term and long-term survival of patients with cancer cachexia. The CTI is a compound index composed of the inflammatory index (CRP) and IR index (TyG). First of all, the CTI index we constructed can reflect not only the level of inflammation but also the state of insulin resistance, which is better than the index alone. Secondly, we also compared the prognostic value of CTI with single inflammatory index and insulin resistance index in cancer patients with cachexia. We found that CTI is better than CRP or TyG alone [Additional file 7]. Lee et al. found that subjects with elevated hs-CRP levels or IR had significantly higher cancer-related mortality [46]. The system inflammation response, as evidenced by elevated CRP levels, is important in the progression of many common solid tumors [47]. Both the primary tumor itself and the related inflammatory response are the cause of cytokine production, and the production of CRP will also increase [24]. Systemic inflammation has now been incorporated into the definition of cachexia as “complex metabolic syndrome associated with underlying diseases characterized by muscle loss with or without fat loss.” Epidemiological studies showed that CRP is correlated with the increased risk of malignant tumors, anorexia-cachexia syndrome, and poor prognosis, including tumor size, tumor recurrence, lymph node metastasis, and distant metastasis [48, 49]. The TyG index is associated with occurrence and progression of cancer [35–37]. Lipotoxicity and glucotoxicity play important roles in the regulation of IR, as reflected by the TyG index. The increased demand for glucose in cancer cells which can cause hypoglycemia, increasing compensatory hormone signals, growth hormones, epinephrine, or glucagon. Hyperinsulinemia itself can induce the increasing production of inflammatory cytokines, thus promoting the IR [30, 50]. An increase in insulin concentration caused by IR may have mitogenic and anti-apoptotic effects [51] and stimulate cell cycle progression in cancer cells [52]. Prolonged hyperinsulinemia may also lead to an increase in free or bioactive IGF-1 levels, which promotes signaling pathways conducive to tumor development [53]. Inflammatory cytokines, including TNF-α, IL-6, and prostaglandin E2, may promote the development of breast cancer by promoting cell proliferation and cell cycle progression [53, 54]. Systemic inflammation is an indicator of the cancer development. Inflammation is the main driving force of metabolic changes in cancer [8]. Persistent inflammatory mediators in cancer patients can stimulate cancer cachexia, which in turn promotes IR [28, 29]. Activation of IR can promote the PI3K/Akt/mTOR and MAP/ERK kinase pathways, eventually leading to cell proliferation, migration, and inhibition of apoptosis [55].

After grouping the patients based on the CTI, we found that, on average, patients with a high CTI were older. We also found that patients with advanced stage cancer cachexia or diabetes had higher CTI values. Older adults with cancer experience higher levels of inflammation and IR than those without cancer. Reduced physical activity and muscle load are key variables affecting skeletal muscle mass and body composition during aging [56]. Cancer cachexia is very common in older adults with cancer and becomes more evident as the disease progresses [57]. Patients with advanced cancer tend to develop cachexia, largely due to long-term malnutrition. Low-grade inflammation is a feature in patients with T2D. Heart disease, metabolic syndrome, and T2D all have one thing in common: inflammation leads to an increase in the concentration of circulating cytokines [58]. IR is an important component of the metabolic syndrome and precedes the secretion of glucagon. The morbidity and mortality of patients with IR have increased, mainly owing to cardiovascular diseases and T2D [59, 60]. Patients with diabetes have impaired or absent insulin secretion and IR [61]. In our study, we found that patients with cancer cachexia and diabetes had a higher CTI, which may be associated with high inflammation and IR in this population.

We also found that patients with high CTI were less active and more malnourished. Thus, we hypothesized that the activity and nutritional status of cancer patients with cachexia are important factors in the poor prognosis of CTI. These results are consistent with our hypothesis that the proportion of patients with activity and malnutrition is higher. Studies have found that physical activity can reduce the risk of colorectal cancer by reducing IR and inflammation [62]. Michael et al. found that increased activity could effectively reduce obesity and improve glucose tolerance and IR [63]. Activity is associated with inflammation and IR, which can mediate poor prognosis in patients with a high CTI. It is well known that cancer cachexia is associated with weight loss, sarcopenia, and low BMI. Anorexia, or compensatory loss of food intake, is a major contributor to the development of cachexia, which is often caused by inflammation. Insulin levels are decreased in patients with cancer with severe malnutrition or weight loss [20]. When patients have long-term inflammation or IR, food intake decreases, leading to malnutrition. Clearly, malnutrition can also mediate the poor prognosis indicated by the CTI.

Our subgroup analysis showed that the CTI was associated with surgery and radiotherapy. Peng et al. found that myasthenia is associated with poor prognosis after surgery for pancreatic cancer [64]. Similarly, Sheetz et al. found that core muscle atrophy was associated with reduced survival after resection in patients with esophageal cancer [65]. Therefore, we hypothesize that the interaction between CTI and surgery may be related to muscle loss, rapid weight loss, or reduced endurance against surgical shocks in patients with low BMI. Successful chemotherapy or radiotherapy restores balance by reactivating immune surveillance, usually by increasing the immunogenicity of cancer cells, releasing risk-related molecular patterns, and/or depleting immunosuppressive white blood cells, such as bone marrow-derived suppressor cells and regulatory T cells, from the tumor bed [66, 67]. Indeed, when a tumor is eliminated, the levels of inflammation and IR in the body decrease. Importantly, we also found that the CTI was positively associated with 90-day and 180-day mortality rates in patients with cancer cachexia. The CTI is related to short-term survival outcomes of patients with cancer cachexia. High levels of inflammation and IR may aggravate the poor prognosis of patients.

This study had some limitations. First, there was a lack of sufficiently detailed data to study the potentially important differences between tumor subtypes (such as breast cancer in terms of receptor status, microsatellite stability, and unstable colorectal cancer). Second, this was a cross-sectional study that only analyzed the data and information collected before treatment and lacked longitudinal change analysis; the time correlation between the CTI and the prognosis of patients with cancer cachexia could not be evaluated. Third, the CTI may reflect tumor heterogeneity in patients with different tumor types. Finally, the prognostic value of the CTI in patients with cancer cachexia needs to be further validated in other cohorts.

## Conclusions

In conclusion, our study is the first to validate the prognostic value of the CTI, an index related to inflammation and IR, in patients with cancer cachexia. The CTI can predict short- and long-term survival outcomes in patients with cancer cachexia. Patients with cancer cachexia and a high CTI had worse OS. In addition, CTI was positively associated with 90-day and 180-day mortality. In clinical practice, the development and use of the CTI can not only reflect inflammation and IR status but also predict the survival outcome of patients. Thus, the CTI is expected to become a practical clinical prognostic indicator.

### Supplementary Information

**Additional file 1. **Flowchart of patient selection for this study.

**Additional file 2. **The optimal cut-off values of CTI in patients with cancer cachexia.

**Additional file 3. **The cumulative survival curves of CTI in the different cohorts of patients with cancer cachexia.

**Additional file 4. **Correlation between CTI and components (CRP and TyG).

**Additional file 5. **Sensitivity analysis*.

**Additional file 6. **Logistic regression analysis.

**Additional file 7. **The prognostic AUC of CTI, CRP, and TyG in patients with cancer cachexia.

## Data Availability

The datasets used and/or analysed during the current study available from the corresponding author on reasonable request.
