# Supplementary figures and images for: Systemic inflammation and insulin resistance-related indicator predicts poor outcome in patients with cancer cachexia
Source: Cancer Metab. 2024 Jan 25;12:3. doi: 10.1186/s40170-024-00332-8 (PMC10809764; doi:10.1186/s40170-024-00332-8)

# Additional file 1 Flowchart of patient selection for this study.


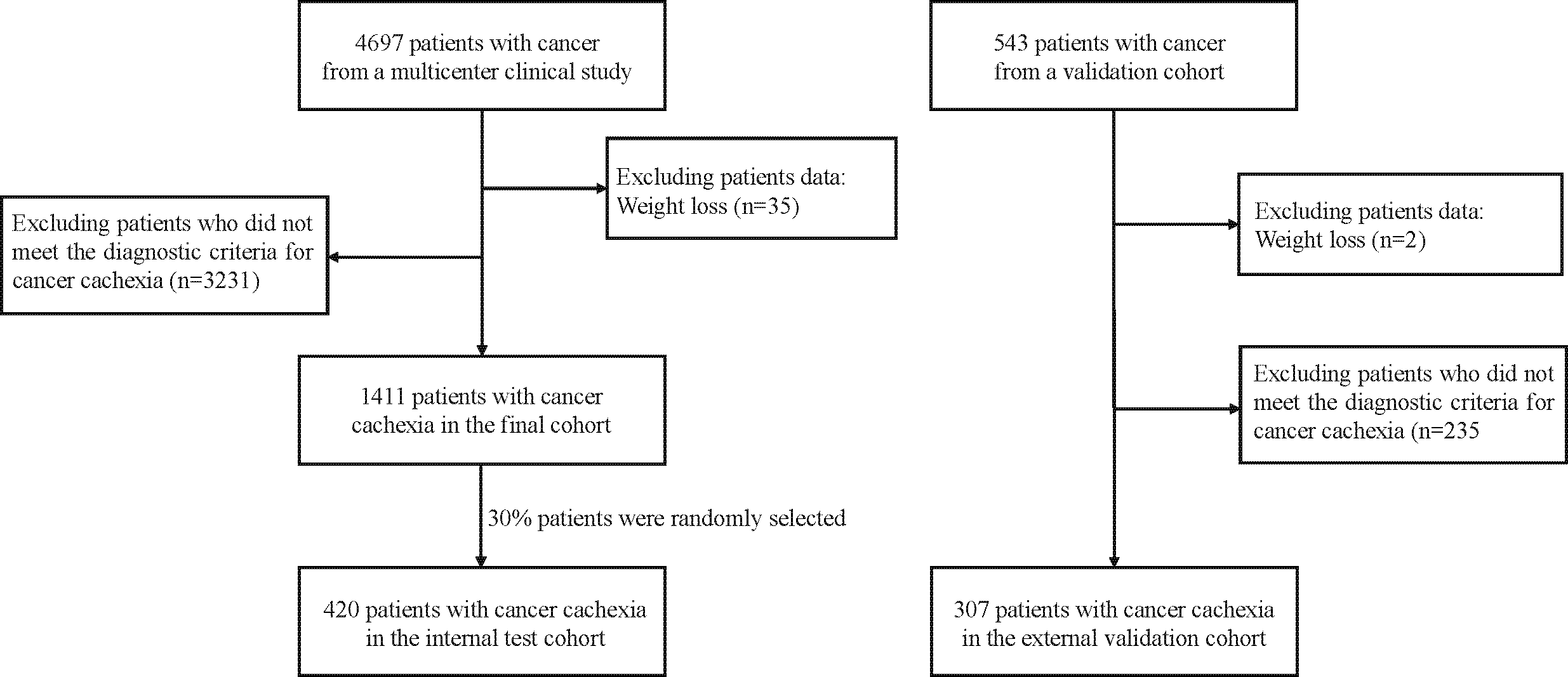

Supplement: Supplementary file 1 — Additional file 1. Flowchart of patient selection for this study. [file 40170_2024_332_MOESM1_ESM.docx]
