# Supplementary material for: Systemic inflammation and insulin resistance-related indicator predicts poor outcome in patients with cancer cachexia
Source: Cancer Metab. 2024 Jan 25;12:3. doi: 10.1186/s40170-024-00332-8 (PMC10809764; doi:10.1186/s40170-024-00332-8)
Supplement: Supplementary file 2 — Additional file 2. The optimal cut-off values of CTI in patients with cancer cachexia. [file 40170_2024_332_MOESM2_ESM.docx]

**Additional file 2 The optimal cut-off values of CTI in patients with cancer cachexia.**


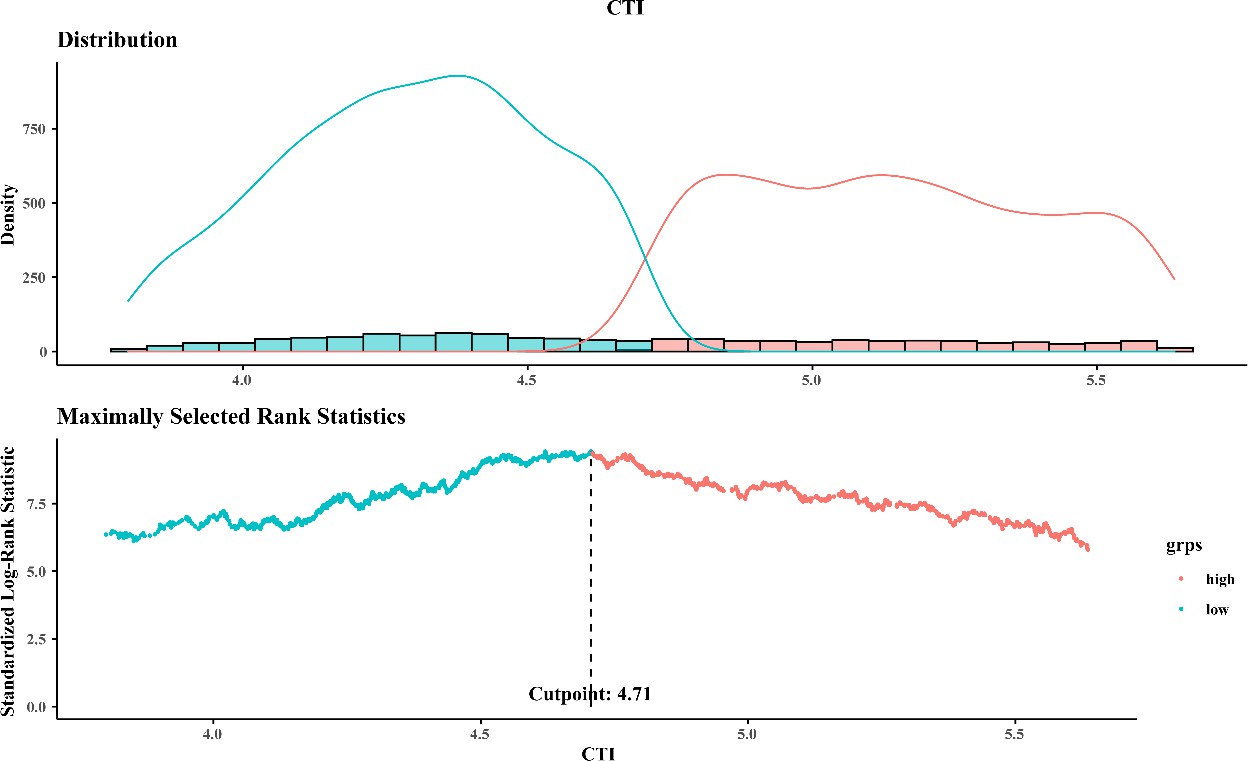


Notes: CTI, C-reactive protein-triglyceride glucose index.
