# Supplementary material for: Systemic inflammation and insulin resistance-related indicator predicts poor outcome in patients with cancer cachexia
Source: Cancer Metab. 2024 Jan 25;12:3. doi: 10.1186/s40170-024-00332-8 (PMC10809764; doi:10.1186/s40170-024-00332-8)
Supplement: Supplementary file 3 — Additional file 3. The cumulative survival curves of CTI in the different cohorts of patients with cancer cachexia. [file 40170_2024_332_MOESM3_ESM.docx]

#
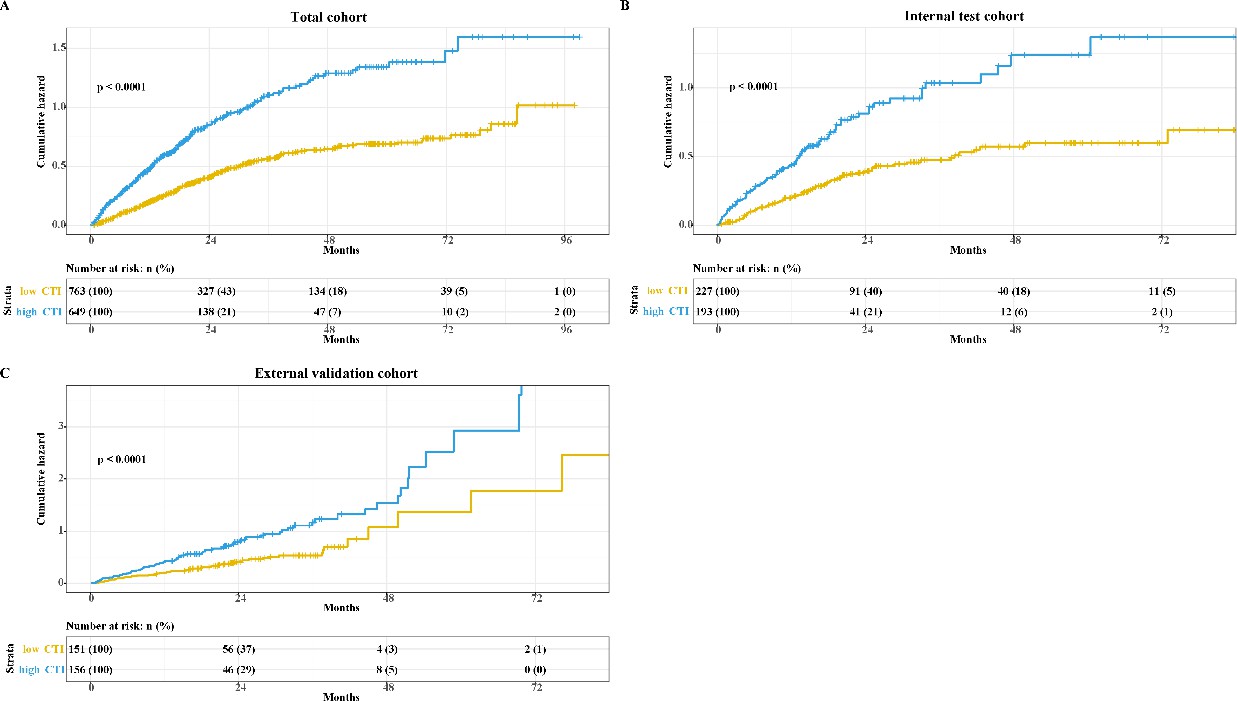
Additional file 3 The cumulative survival curves of CTI in the different cohorts of patients with cancer cachexia.

(A) Total cohort; (B) Internal validation cohort; (C) External validation cohort. The "yellow line" represents patients with cancer cachexia with low CTI, and the "blue line" represents patients with cancer cachexia with high CTI.

Notes: CTI, C-reactive protein-triglyceride glucose index.
