# Supplementary material for: Systemic inflammation and insulin resistance-related indicator predicts poor outcome in patients with cancer cachexia
Source: Cancer Metab. 2024 Jan 25;12:3. doi: 10.1186/s40170-024-00332-8 (PMC10809764; doi:10.1186/s40170-024-00332-8)
Supplement: Supplementary file 5 — Additional file 5. Sensitivity analysis*. [file 40170_2024_332_MOESM5_ESM.docx]

# Additional file 5 Sensitivity analysis*

| Variables | OS (model 0) |  |  | OS (model 3) |  |
| --- | --- | --- | --- | --- | --- |
|  | Crude HR(95%CI) | Crude P |  | Adjusted HR(95%CI) | Adjusted P |
| as continues (per SD) | 1.37 (1.27-1.49) | <0.001 |  | 1.17 (1.07-1.27) | 0.001 |
| By cut-off |  |  |  |  |  |
| CTI<4.71 | ref. |  |  | ref. |  |
| CTI≥4.71 | 1.85 (1.57-2.20) | <0.001 |  | 1.33 (1.10-1.60) | 0.003 |
| By Interquartile |  |  |  |  |  |
| Q1 | ref. |  |  | ref. |  |
| Q2 | 1.30 (1.01-1.68) | 0.041 |  | 1.26 (0.97-1.63) | 0.089 |
| Q3 | 1.88 (1.48-2.40) | <0.001 |  | 1.43 (1.11-1.85) | 0.006 |
| Q4 | 2.32 (1.81-2.96) | <0.001 |  | 1.53 (1.17-1.99) | 0.002 |
| p for trend |  | <0.001 |  |  | 0.001 |
| By tertiles |  |  |  |  |  |
| T1 | ref. |  |  | ref. |  |
| T2 | 1.53 (1.24-1.89) | <0.001 |  | 1.36 (1.09-1.69) | 0.007 |
| T3 | 2.10 (1.70-2.60) | <0.001 |  | 1.47 (1.17-1.84) | 0.001 |
| p for trend |  | <0.001 |  |  | 0.001 |

Notes: * The sensitivity analysis was to exclude patients who died within 3 months. OS, overall survival; HR, hazards ratio; CI, confidence interval; CTI, CRP-TyG index; CRP, C-reactive protein; TyG: triglyceride-glucose index; BMI: body mass index; KPS, karnofsky performance status; EORTC QLQ-C30, The European Organization for Research and Treatment of Cancer (EORTC), Quality of Life Questionnaire-Core 30 (QLQ-C30); ECOG PS: eastern cooperative oncology group performance status; PGSGA, Patient Generated Subjective Global Assessment; TSF, triceps skinfold thickness.

Model 0: Unadjusted.

Model 3: Adjusted for age, sex, BMI, tumor stage, tumor types, KPS, surgery, chemotherapy, radiotherapy, smoking status, alcohol consumption, KPS, EORTC QLQ-C30, ECOG PS, PGSGA, diabetes, hypertension, coronary heart disease, and TSF.
