# Supplementary material for: Systemic inflammation and insulin resistance-related indicator predicts poor outcome in patients with cancer cachexia
Source: Cancer Metab. 2024 Jan 25;12:3. doi: 10.1186/s40170-024-00332-8 (PMC10809764; doi:10.1186/s40170-024-00332-8)
Supplement: Supplementary file 6 — Additional file 6. Logistic regression analysis. [file 40170_2024_332_MOESM6_ESM.docx]

# Additional file 6 Logistic regression analysis.

| variables | Crude OR(95%CI)* | Crude P | Adjusted OR(95%CI)# | Adjusted P |
| --- | --- | --- | --- | --- |
| **90-day mortality** | | | | |
| CTI<4.71 | ref. |  | ref. |  |
| CTI≥4.71 | 4.56 (2.97-7.22) | <0.001 | 2.48 (1.52-4.14) | <0.001 |
| **180-day mortality** | | | | |
| CTI<4.71 | ref. |  | ref. |  |
| CTI≥4.71 | 3.04 (2.23-4.17) | <0.001 | 1.77 (1.24-2.55) | <0.001 |

Notes: OR, odd ratio; CI, confidence interval; CTI, CRP-TyG index; CRP, C-reactive protein; TyG: triglyceride-glucose index; BMI: body mass index; KPS, karnofsky performance status; EORTC QLQ-C30, The European Organization for Research and Treatment of Cancer (EORTC), Quality of Life Questionnaire- Core 30 (QLQ-C30); ECOG PS: eastern cooperative oncology group performance status; PGSGA, Patient Generated Subjective Global Assessment; TSF, triceps skinfold thickness.

*Model 0: Unadjusted.

#Model 3: Adjusted for age, sex, BMI, tumor stage, tumor types, KPS, surgery, chemotherapy, radiotherapy, smoking status, alcohol consumption, KPS, EORTC QLQ-C30, ECOG PS, PGSGA, diabetes, hypertension, coronary heart disease, and TSF.
