# Supplementary material for: Systemic inflammation and insulin resistance-related indicator predicts poor outcome in patients with cancer cachexia
Source: Cancer Metab. 2024 Jan 25;12:3. doi: 10.1186/s40170-024-00332-8 (PMC10809764; doi:10.1186/s40170-024-00332-8)
Supplement: Supplementary file 7 — Additional file 7. The prognostic AUC of CTI, CRP, and TyG in patients with cancer cachexia. [file 40170_2024_332_MOESM7_ESM.docx]

# Additional file 7 The prognostic AUC of CTI, CRP, and TyG in patients with cancer cachexia


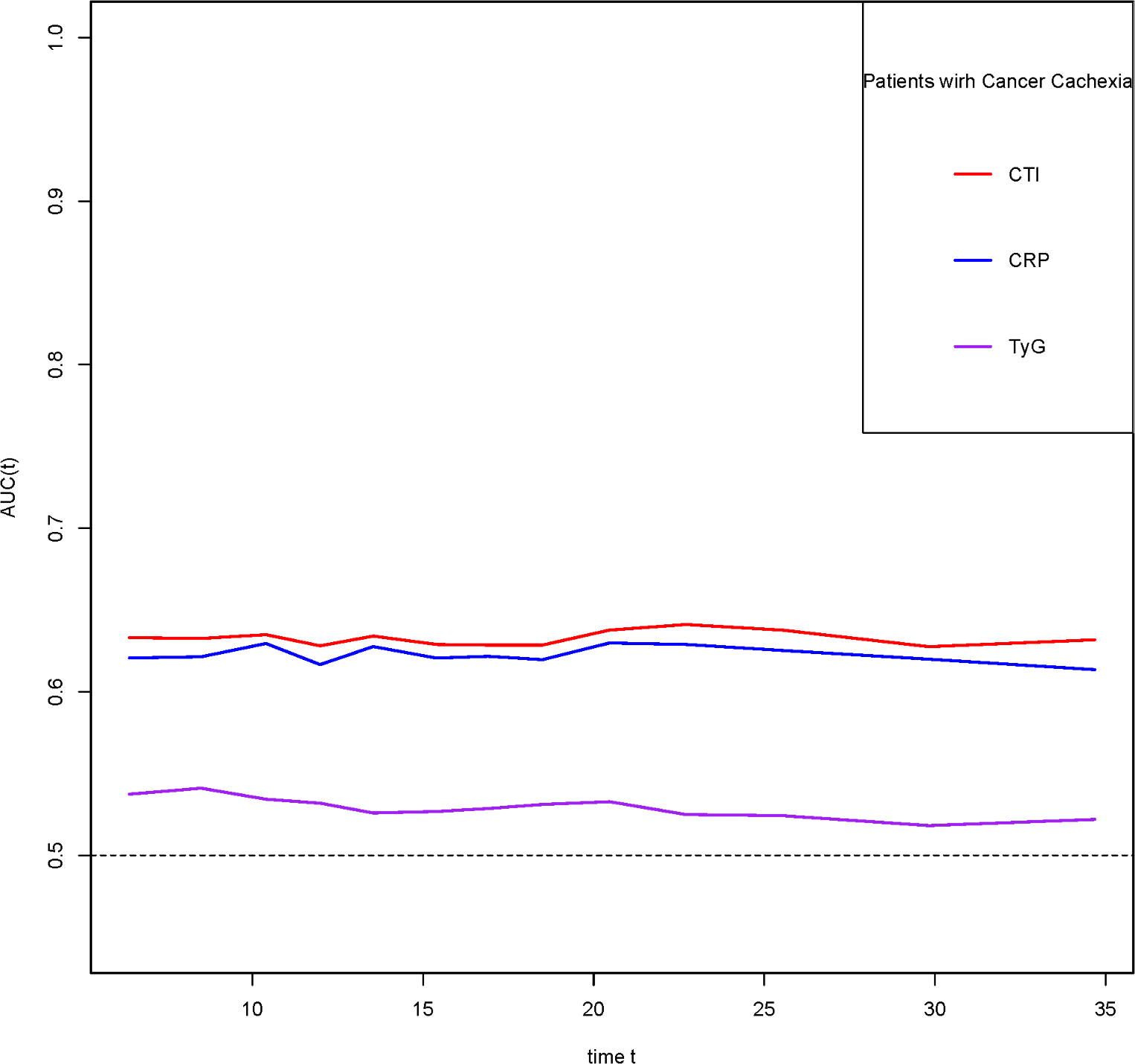


Notes: AUC, area under curve; CTI, C-reactive protein-triglyceride glucose index; CRP: C-reactive protein; TyG: triglyceride-glucose index.
